# Speech-based respiratory diagnostics: A study on COVID-19 detection with machine learning

**DOI:** 10.1371/journal.pone.0332146

**Published:** 2025-11-21

**Authors:** Gaurav Datkhile, Pramod H. Kachare, Sandeep B. Sangle, Ibrahim Al-Shourbaji, Abdoh Jabbari, Raimund Kirner, Abdalla Alameen

**Affiliations:** 1 Department of Computer Science Engineering, Ramrao Adik Institute of Technology, Navi Mumbai, India; 2 Department of Electrical and Electronics Engineering, Jazan University, Jazan, Saudi Arabia; 3 Department of Computer Science, University of Hertfordshire, Hatfield, United Kingdom; 4 Department of Computer Engineering and Information, College of Engineering in Wadi Alddawasir, Prince Sattam Bin Abdulaziz University, Wadi Alddawasir, Saudi Arabia; Manipal Academy of Higher Education, INDIA

## Abstract

Respiratory sound analysis has emerged as a promising approach for detecting and diagnosing respiratory diseases, including COVID-19. This study investigates using OpenSMILE features for COVID-19 detection using vowel speech sounds /a/, /e/, and /o/ from the COSWARA dataset. OpenSMILE facilitates the extraction of audio and functional features, which are then classified using various machine learning algorithms. Multiple ML classifiers Random Forest (RF), Support Vector Machine, Decision Tree, and Artificial Neural Network are evaluated. To enhance classification performance, five distinct feature selection techniques were applied: ANOVA, chi-square, Information Gain, ReliefF, and Gini index. Among these, ANOVA-based selection yielded the most consistent results across classifiers and vowel sounds. Among the models evaluated, the RF classifier achieved the highest accuracies of 76.47% for vowel /a/ and 75.54% for vowels /a/ and /o/, respectively, when combined with ANOVA-selected features (155, 163, and 161 features). To statistically assess model and feature selection performances, the Friedman test was conducted across classifiers and feature selection techniques. Results confirmed the significance of Random Forest and ANOVA as robust combinations. This research contributes to developing accessible, scalable, and non-invasive diagnostic tools, enhancing the potential of telemedicine and remote healthcare systems for the early detection of respiratory diseases.

## 1 Introduction

Recently, there has been an increasing emphasis on developing affordable, rapid, and scalable techniques for the detection of respiratory diseases. Diagnostic tools such as chest X-rays and CT scans are commonly used for respiratory conditions like COVID-19, as they reveal critical anatomical features of the lungs [1,2]. However, these techniques expose patients to harmful radiation and incur high costs; instead, monitoring speech signals is more effective. The classification and diagnosis of COVID-19 based on respiratory sound analysis is an area that has attracted a lot of interest from healthcare researchers and scientists. This method is non-invasive, radiation-free, and poses no health risk to patients. Researchers have proposed diverse approaches encompassing signal processing, ML, and Deep Learning (DL) techniques that are employed to tackle this real-world problem [2–4]. In the application of signal processing techniques, these sound signals, though invisible, can be effectively denoised and analyzed to extract meaningful features. This enables the identification of abnormal respiratory patterns associated with diseases like COVID-19. These approaches enable the development of scalable, contactless, and cost-effective diagnostic tools, making respiratory sound analysis a compelling alternative to conventional imaging for COVID-19 detection. Different respiratory sounds, such as speech, cough, breathing, and lung sounds, are used by doctors and technicians to diagnose the disease [5]. Human respiratory sounds have tremendous potential for early disease diagnosis and low-cost treatments. In this study, speech-based machine learning models were used for COVID-19 detection. Several methods have been proposed in the literature to extract relevant features from clean respiratory sound signals to detect COVID-19 [4,6–9].

Diverse interferences can affect these signals, such as microphone contact, muscle contractions and expansions, noises from other medical devices, speech, mobile devices, and other sources. Different kinds of noise interference may contribute to misdiagnosis. Hence, effectively eliminating or reducing the effect of noise in the detection of respiratory sounds is crucial for accurate diagnosis and efficient prevention and treatment of diseases.

In [10], Mel spectrogram and GFCC features are proposed for the detection of COVID-19. For data augmentation, the authors used color transformation. Various noise levels were added to the features. The deep shuffleNet model was used for classification. Authors reported 87.8% accuracy for mel spectrogram features added with combined augmented data.

Audio enhancement techniques with multitasking and cold cascade methods are studied in [11] for the detection of COVID-19. This work used environmental noises with different SNR levels for training and testing. The highest Area under the curve (AUC) was observed to be 81.73% at 25 dB SNR using the multi-tasking process, and it is 71.1% for 0 dB SNR value.

In [12], the Environmental Sound Classification 50 (ESC-50) dataset is used to find COVID-19. They created and evaluated a phone app using 76 typical cough sounds, 102 of bronchitis, 131 of pertussis, and 48 COVID-19 cough sounds. The authors used Mel-frequency cepstral coefficients (MFCC) M × N feature matrix concatenated with the highest 2 projection vectors of its Principal Component Analysis (PCA). Deep learning and machine learning algorithms are used for classification. They reported an accuracy rate of 92.64% in correctly identifying the different types of sounds. The authors in [6,7] proposed transfer learning-based deep neural network (DNN) methods for COVID-19 detection using speech, cough, and breath sounds. The ResNet50 classifier achieved accuracy rates of 97%, 91%, and 87%, respectively, using 1,171 respiratory sound samples. Features such as MFCC, ZCR, and kurtosis were extracted from the dataset.

In [4], 1,040 cough samples, including COVID-19 and non-COVID-19 cases, were obtained from the Dicova dataset for log spectrogram feature extraction. A deep neural network with fully connected layers was pruned based on the Lottery Ticket Hypothesis (LTH), with the VGG-13 architecture fine-tuned for this application. The model evaluation showed an AUC of 0.783 and a sensitivity of 80.49%, trained using binary cross-entropy and focal losses, along with data augmentation.

In [8], the authors evaluated an ensemble model by combining the ICBHI with the Coswara speech, cough, and breathing datasets. Approximately 110 samples from the COVID-19 positive and negative classes were used for respiratory analysis. The model has four base deep networks: Attention-based CNN (A-CRNN), attention-based Bidirectional Long Short-Term Memory (A-BiLSTM), attention-based Bidirectional Gated Recurrent Unit (A-BiGRU), and CNN. The Particle Swarm Optimization (PSO) algorithm optimizes the training parameters. The ensemble mechanism integrates the outputs by averaging the probability predictions of each class. The accuracy reported for cough sounds using the A-BiGRU model is 98.25 Similarly, the ensemble model achieves accuracy rates of 93% and 92.40% for breath and speech sounds, respectively. In [9], the authors used vowel sounds (/a/, /e/, /o/) from the COSWARA dataset. They reported an accuracy of 97.07% using 205 testing samples and 822 training samples. They used a smaller subset of the Coswara dataset, A limitation in [9], approach is the lack of explicit validation, as they did not use a dedicated validation set during training, potentially leading to overfitting.

In this work, our aim is to automatically classify patients’ respiratory sound patterns for diagnosing COVID-19 disease in noisy environment. To achieve this goal, OpenSMILE is used to extract audio and functional features, and a set of ML models are used to classify COVID-19 and healthy vowel sounds. The major contributions of this work can be summarized as follows:

- Identifying the significant Speech feature bands for COVID-19 detection in the early stages.
- Investigating the performance of Feature selection using different ML algorithms.
- Comparative classification performance analysis for COVID-19 and Non COVID-19 detection.

The rest of the paper is organized as follows: Sect 2 discusses a brief description of the methods used in the proposed COVID-19 detection technique. Sect 3 deals with various experimental results obtained by the proposed method, and finally, the inferences and conclusions drawn from the obtained results are discussed in Sect 4.

## 2 Methodology

**Fig 1:**
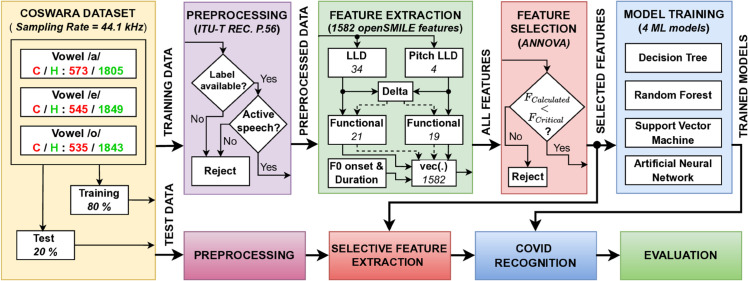
System block diagram.

This section introduces the proposed system. Fig 1 presents the main phases of the introduced system for COVID-19 detection from vowel speech.

### 2.1 COSWARA dataset

**Fig 2:**
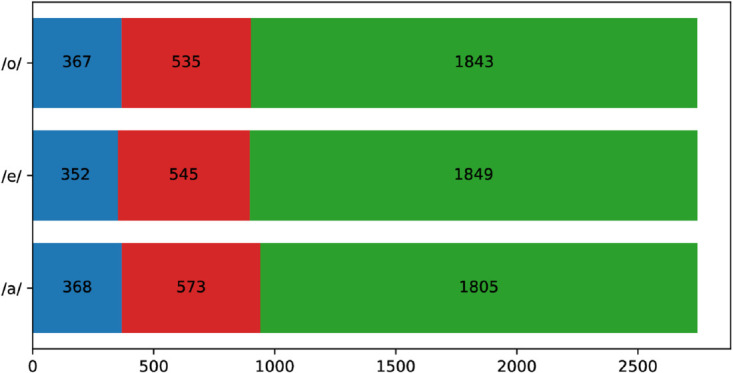
A summary of noisy (blue), COVID-19 (red), and healthy (green) recordings in the COSWARA dataset.

In the present study, the COSWARA dataset for COVID-19 vowel speech has been utilized. The COSWARA dataset was created by the Indian Institute of Science, Bengaluru, [13] (accessed on July 2024). This dataset is freely available on the GitHub repository. It includes recordings of shallow cough, deep and shallow breath, vowel-A, vowel-E, and vowel-O sounds from COVID-19 and healthy subjects. In this work, the results are represented using all these sounds to classify COVID-19 from healthy recordings. All the raw recordings were available at a sampling frequency of 44.1 kHz. The dataset contains approximately 2700 sound recordings of heavy cough, shallow cough, deep breath, and shallow breath, vowel-*/a/*, vowel-*/e/*, and vowel-*/o/*. Fig 2 provides a summary of the distribution of recordings. The noisy rejected recordings are represented in ; COVID-19 recordings are shown in Red, and healthy recordings are depicted in Green. The preprocessing details are provided below.

### 2.2 Pre-processing

Preprocessing is a crucial step in the analysis of speech sound signals, particularly for tasks such as speech recognition, speaker identification, and medical diagnosis. Preprocessing techniques like filtering help remove this noise, improving the quality of the signal for analysis. Speech signals can vary widely in amplitude and energy, depending on the speaker’s voice strength, distance from the microphone, and other factors. Normalization ensures that the amplitude or volume of the signal is consistent across different recordings, which allows for better comparison and analysis. In this study, the Active Speech Level (ASL) is defined in ITU-T P.56, which is a standard developed by the International Telecommunication Union (ITU) to measure the amplitude or loudness of speech signals [14]. ITU-T P.56 introduces an algorithm that segments speech into frames and calculates the energy in each frame. The frames with energy above a certain threshold are considered active speech, while the others are treated as silence. This threshold-based method ensures that the measurement is robust even in the presence of noise. ASL is used as a preprocessing step to normalize the amplitude of speech data, making it consistent across different recordings. This helps reduce variability due to differences in loudness between speakers or recording conditions, which in turn improves the performance [15].

**Fig 3:**
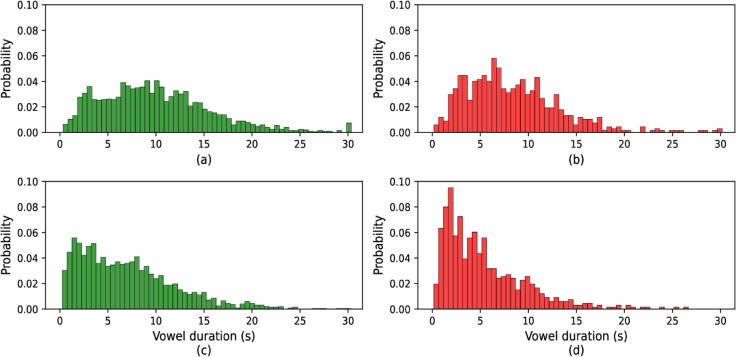
Histogram of vowel /a/ duration: (a) original COVID, (b) original healthy, (c) active speech processed COVID, and (d) active speech processed healthy.

**Fig 4:**
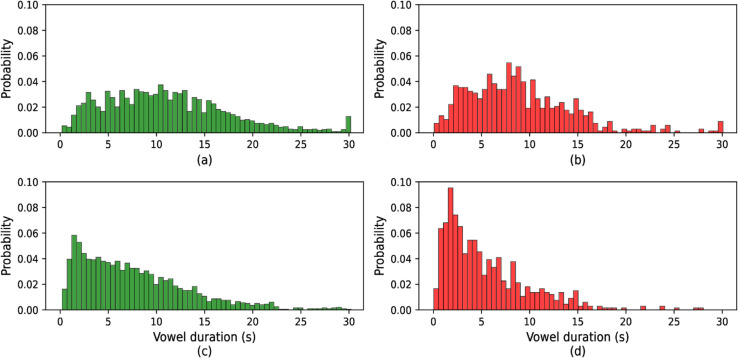
Histogram of vowel /e/ duration: (a) original COVID, (b) original healthy, (c) active speech processed COVID, and (d) active speech processed healthy.

**Fig 5:**
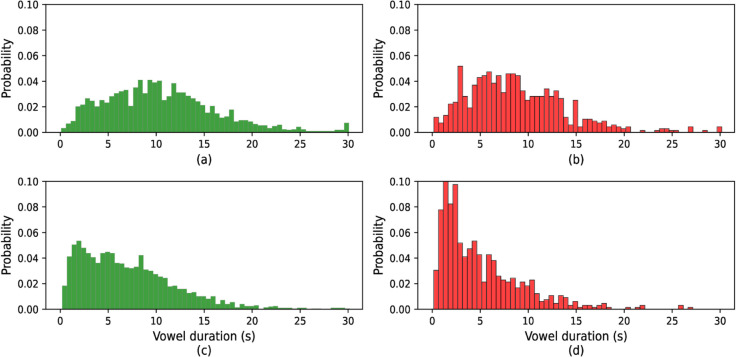
Histogram of vowel /o/ duration: (a) original COVID, (b) original healthy, (c) active speech processed COVID, and (d) active speech processed healthy.

The Figs 3, 4, and 5 display the probability distributions of vowel /a/, /e/, and /o/ sound durations, respectively, for both healthy individuals and those with COVID-19. These figures provide a comparative view of how the durations of these vowel sounds differ between the two groups. The distribution for each vowel in the healthy group likely follows a distinct pattern, while the COVID-19 group may exhibit noticeable variations, suggesting that the infection influences speech characteristics, particularly vowel duration. This analysis is crucial in identifying potential markers in speech patterns that could be associated with health conditions like COVID-19.

For the original COVID conditions (Figs 3, 4, and 5 part a), the distributions exhibit a wider spread with longer tail durations, indicating a tendency for COVID patients to produce more extended vowel sounds. This contrasts with the original healthy distributions (part b), which are more concentrated around shorter durations, showing a more typical speech pattern with less variance.

After active speech processing, both COVID and healthy conditions (Figs 3, 4, and 5 parts c and d, respectively) show more uniform distributions with reduced spread. The processing reduces the longer vowel durations seen in COVID patients, bringing their distributions closer to the healthy subjects’ processed data. However, even after processing, the COVID distributions tend to retain slightly longer durations compared to their healthy counterparts, suggesting that speech abnormalities persist to some extent despite active speech processing. Overall, these figures illustrate how vowel duration in speech is influenced by both health conditions and signal processing, with COVID speech generally showing longer and more varied vowel durations compared to healthy speech, though processing mitigates some of these differences.

### 2.3 OpenSMILE feature

In this study, acoustic feature extraction is conducted using the OpenSMILE (Open-source Speech and Music Interpretation by Large-space Extraction) toolkit, which is widely adopted in speech processing and paralinguistic research. It resulted in a 1582-dimensional feature vector per recording. In OpenSMILE, features included are 34 Low-Level Descriptors (LLDs) such as energy, zero-crossing rate, spectral centroid, spectral flux, spectral roll-off, Mel-Frequency Cepstral Coefficients (MFCCs), and voice-related features like jitter and shimmer. 4 Pitch-related LLDs, including fundamental frequency (f0), voicing probability, harmonics-to-noise ratio (HNR), and pitch confidence.

A comprehensive set of statistical functionals (e.g., mean, standard deviation, skewness, kurtosis, percentiles, minima, maxima, linear regression coefficients) was computed for each LLD, resulting in fixed-length representations suitable for machine learning classification tasks.

These features were selected to capture the speech signal’s short-term temporal dynamics and long-term statistical trends. Such acoustic cues are especially relevant in COVID-19 detection, where subtle impairments in respiratory and phonatory systems may lead to measurable changes in voice quality, pitch stability, and spectral structure.

### 2.4 Feature selection

Feature selection is the process of choosing a subset of the most relevant features from a larger set, focusing on those that significantly influence the outcome. While a straightforward approach might involve evaluating model performance across all possible feature combinations and selecting the subset that yields the best results, this method is inefficient when dealing with numerical input features and categorical output. Instead, it is essential to identify features that are highly dependent on the response variable.

One method for feature selection highlighted in the paper is the ANOVA F-statistic method. Analysis of Variance (ANOVA) is a parametric statistical hypothesis test used to determine whether the means of two or more data samples originate from the same distribution. The ANOVA method uses a correlation technique to eliminate features that are independent of the target variable, thereby reducing computational complexity and time while mitigating the curse of dimensionality. This approach ultimately enhances accuracy. The ANOVA approach ranks features by calculating the ratio of variances between and within groups.

This ratio indicates the strength of the relationship between the $\lambda^{th}$ feature and the group variables. The F-value for the $\lambda^{th}$ g-gap dipeptide in two benchmark datasets can be calculated using the following equation:

$$F(\lambda )=\frac{s_{B}^{2}(\lambda )}{s_{W}^{2}(\lambda )}$$ (1)

where $s_{B}^{2}(\lambda )$ is the Between-group variance and $s_{W}^{2}(\lambda )$ is the Within-group variance for the feature (λ).

#### 2.4.1 Between-group variance.

This measures the variance of the feature (λ) among the different groups (target classes). A higher variance between groups implies that the feature is useful for distinguishing between the groups.

$$s_{B}^{2}(\lambda )=\frac{1}{k-1}\sum_{i=1}^{k}n_{i}({\bar{x}}_{i}-\bar{x}{)}^{2}$$ (2)

where,

- k is the number of groups (classes),
- *n*_*i*_ is the number of observations in group i,
- ${\bar{x}}_{i}$ is the mean of group i,
- $\bar{x}$ is the overall mean of the data (grand mean).

#### 2.4.2 Within-group variance.

This measures the variance of the feature (λ) within each group. Lower within-group variance indicates that the feature values are consistent within each class.

$$s_{W}^{2}(\lambda )=\frac{1}{N-k}\sum_{i=1}^{k}\sum_{j=1}^{n_{i}}n_{i}(x_{ij}-{\bar{x}}_{i}{)}^{2}$$ (3)

where,

- N is the number of observations across all groups,
- *x*_*ij*_ is the individual data point j in group i,
- ${\bar{x}}_{i}$ is the mean of group i.

### 2.5 ML models

The primary goal of ML methods is to identify the function that maps input features to the output class. In this work, we use the COSWARA dataset, which contains COVID-19 vowel speech data. Decision Trees (DT), RF, Support Vector Machines (SVM), and ANN are employed as mapping functions in this study.

#### 2.5.1 Decision Tree (DT).

The DT model identifies patterns in the training dataset and creates its own splitting rules for classification purposes. The Gini Index serves as the cost function for constructing the decision tree in the COSWARA dataset, which contains COVID-19 vowel speech data. This index is instrumental in generating rules from the training data. The Gini Index is calculated as follows:

$$Gini(t)=1-\sum_{i=0}^{c-1}[p(i|t){]}^{2}$$ (4)

The Gini score provides insight into the quality of a split by assessing how mixed the classes are within the two labeled groups created by the split. A perfect separation yields a Gini score of 0, while a split resulting in an equal distribution of classes (50/50) reflects the worst-case scenario. The Gini Index is computed for each node, guiding the data splitting in the binary tree. This process is repeated recursively to build the tree. However, a challenge with Decision Trees is the potential for overfitting or underfitting, as the model may “memorize" the training set rather than generalize well. To mitigate this issue, ensemble learning techniques, such as Random Forests and Gradient Boosted Trees, can be employed. These methods enhance the model’s performance and robustness, particularly when applied to complex datasets like COSWARA.

#### 2.5.2 Random Forest (RF).

RF is an ensemble learning technique designed to improve the accuracy and robustness of classification tasks. It works by constructing multiple DTs and using a majority vote to make final classifications. In this study, RF classifier utilizing the “gini” impurity criterion was employed. The area under the receiver operating characteristic curve (AUC-ROC) served as the performance measure. RF is particularly effective at handling large and complex datasets, managing high-dimensional feature spaces, and providing insights into feature importance. It is renowned due to its ability to maintain high predictive accuracy while minimizing overfitting, making it a widely favored approach across several fields, such as finance, healthcare, and image analysis, among other ML models. It is effective in improving the accuracy and reliability of classification tasks, as demonstrated in this study. Parameters like the number of trees (*n*_*e*_*stimators* = 100) is selected. Using more trees typically improve the overall performance of the model but it requires more computational resources. The GINI index criterion is used to measure the quality of a split. The maximum depth of the tree is kept as None, so nodes are expanded until all leaves are pure. The random state ensures consistent results across runs.

#### 2.5.3 Support Vector Machine (SVM).

The SVM model is a supervised ML METHOD designed to classify data by identifying an optimal hyperplane that maximizes the margin between different classes in an N-dimensional space. This hyperplane serves as the decision boundary, ensuring that the separation between classes is as wide as possible. SVMs are versatile and can handle both linear and non-linear classification tasks by applying different kernel functions. In the context of the COSWARA dataset, which involves analyzing COVID-19 vowel speech data, SVMs are particularly useful due to their ability to manage high-dimensional feature spaces and perform well even with a smaller number of data points. By utilizing an appropriate kernel function, SVMs can effectively capture complex relationships between features in the COSWARA dataset, enabling accurate classification of speech patterns that may indicate COVID-19. This makes SVM a powerful tool for tasks that require precise and reliable classification, such as detecting subtle variations in speech that could be associated with the disease. This model uses the RBF kernel, which can handle nonlinear relationships by mapping the input features into a higher-dimensional space. The C parameter is set to 1 to control the relation between a low error on the training data and minimizing model complexity is set to 10. The gamma parameter is set to a linear scale.

#### 2.5.4 Artificial Neural Network (ANN).

ANNs are computational models that excel in tasks such as learning, generalizing, clustering, and organizing data. They are characterized by their ability to adapt and learn from data through a massively parallel architecture that mimics the human brain’s neural networks. In the context of the COSWARA dataset, which involves analyzing COVID-19 vowel speech data, ANNs are particularly effective due to their capability to capture complex patterns and relationships within the data. The flexibility of ANNs allows them to model non-linear dependencies and subtle variations in speech that may be indicative of COVID-19. By learning from the dataset, ANNs can generalize well to new, unseen data, making them a powerful tool for identifying potential COVID-19 cases based on speech analysis. This adaptability and learning capability make ANNs highly suitable for handling the intricate and varied nature of the COSWARA dataset. The ANN classifier uses one hidden layer containing 64 neurons with a ReLU activation function. ANN model learns using 50 iterations from the training data with a 0.001 learning rate. Adam optimizer used for the model learns from the data by adjusting the weights.

The hyperparameter settings of these models are defined based on their implementations in original works, and they are listed in Table 1.

**Table 1:** Hyperparameter tuning ranges used for grid search.

Classifier	Hyperparameter	Values Tested
DT	Max Depth (max_depth)	None, 10, 20, 30
	Criterion	Gini, Entropy
	Min Samples Split	2, 5, 10
RF	Number of Trees (n_estimators)	50, 100, 200
	Max Depth (max_depth)	None, 10, 20, 30
	Criterion	Gini, Entropy
SVM	Kernel Type	Linear, RBF, Polynomial
	Regularization Parameter (*C*)	0.1, 1, 10
	Gamma (for RBF/Poly)	Scale, 0.01, 0.001
ANN	Hidden Layers	1, 2, 3
	Neurons per Layer	64, 128, 256
	Activation Function	ReLU, Tanh
	Optimizer	Adam, SGD
	Learning Rate	0.001, 0.0001
	Batch Size/Epochs	32/50

### 2.6 Hypothesis

For the proposed hypothesis,

- H0: Positive prediction represents that the person is suffering from COVID-19
- H1: Negative prediction that a person is healthy.

**Fig 6:**
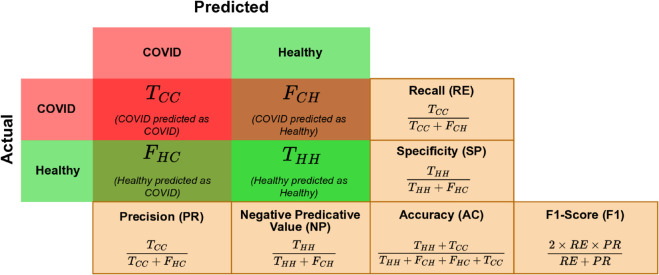
Confusion Matrix for result analysis.

The confusion matrix is used to analyse the desired target and predicted class labels, as shown in Fig 6.

- *T*_*CC*_ : COVID Predicted as Covid
- *T*_*HH*_ : Healthy Predicted as Healthy
- *F*_*CH*_ : Covid Predicted as Healthy
- *F*_*HC*_ Healthy Predicted as Healthy

## 3 Result and discussion

The results of COVID detection without applying feature selection techniques are shown in Table 2. A 5-fold cross-validation strategy is used during feature selection and hyperparameter tuning to ensure robust model evaluation and avoid overfitting. In this approach, the dataset was randomly partitioned into five equal-sized folds. Four folds were used for training for each iteration, and the remaining one was used for validation. This process was repeated five times, ensuring each fold was used once for validation. The final performance metrics were computed by averaging all five folds. This technique provides a reliable estimate of model generalizability and helps mitigate the effects of data imbalance or variability.

**Table 2:** Result analysis of COSWARA dataset without feature selection.

Vowel	Metric	DT	RF	SVM	ANN
/a/	Accuracy	66.66	76.09	75.31	74.00
	F1-Score	66.15	75.24	75.50	73.08
	Precision	65.74	74.42	75.70	72.19
	Recall	66.60	76.09	75.31	72.86
/e/	Accuracy	64.63	74.65	75.15	77.32
	F1-Score	65.22	72.99	78.15	76.33
	Precision	65.89	71.38	81.39	75.32
	Recall	64.63	74.65	75.15	74.88
/o/	Accuracy	65.33	75.92	74.77	74.44
	F1-Score	65.70	76.09	74.99	75.45
	Precision	66.10	76.27	75.22	74.44
	Recall	65.35	75.92	74.77	74.39

The RF achieved the highest accuracy for vowel /a/, /e/and vowel /o/. In terms of F1-score, the SVM classifier outperformed others for all vowels.

The Tables 3 and 4. presents the accuracy and F1 score values of different ML models with the vowel (/a/, /e/, and /o/), using five different feature selection techniques: ANOVA (ANO), Chi-square (*Chi*^2^), Information Gain (IG), ReliefF, and Gini index. Random Forest demonstrates the most consistent performance across all vowels and feature selection methods, with accuracies generally around 75–76%. It indicates that RF is relatively robust and less sensitive to feature selection. ANN models perform moderately, with noticeable improvement when using Gini, *Chi*^2^, and IG. SVM achieves high accuracy (around 75–76%) only when ANOVA is used, but its performance drops drastically with other feature selection methods.

**Table 3:** Accuracy and F1-score comparison across feature selection techniques.

	Model	Accuracy (%)					F1 Score (%)				
		ANO	Chi^2^	IG	ReliefF	Gini	ANO	Chi^2^	IG	ReliefF	Gini
/a/	DT	67.74	68.40	67.80	65.40	67.00	68.12	68.20	67.40	64.90	67.20
	RF	76.47	75.90	76.20	75.20	75.90	75.40	69.60	70.60	65.40	70.00
	SVM	75.96	40.90	42.80	57.60	42.00	77.43	42.10	44.90	60.00	44.00
	ANN	72.86	73.80	74.60	74.60	74.60	72.02	71.50	72.70	72.60	71.80
/e/	DT	70.44	66.54	67.03	65.44	67.03	74.97	66.37	66.79	65.14	66.79
	RF	75.54	75.13	74.05	75.48	75.29	70.66	67.01	69.03	66.28	67.21
	SVM	75.03	48.25	46.24	58.67	46.50	75.36	50.76	48.11	60.90	48.56
	ANN	72.22	73.50	73.95	71.83	73.88	72.13	70.39	70.79	69.67	68.73
/o/	DT	71.44	73.26	71.64	67.42	68.11	74.97	67.81	68.20	66.96	67.62
	RF	75.54	74.79	73.02	74.68	76.37	70.66	69.24	69.62	67.45	70.26
	SVM	74.31	48.37	45.81	61.34	46.96	75.36	50.63	47.67	63.47	48.96
	ANN	73.22	73.08	74.57	72.93	74.95	72.13	70.40	70.19	71.22	71.67

**Table 4:** Precision and recall comparison across feature selection techniques.

	Model	Precision (%)					Recall (%)				
		ANO	chi^2^	IG	ReliefF	Gini	ANO	chi^2^	IG	ReliefF	Gini
/a/	DT	68.55	67.90	67.20	64.50	67.40	67.74	68.40	67.50	65.40	67.00
	RF	74.36	72.50	73.10	74.10	72.40	76.47	75.90	76.20	75.20	75.90
	SVM	77.92	63.70	63.30	64.40	62.50	76.96	40.90	42.80	57.60	42.00
	ANN	71.19	70.70	72.00	71.90	71.30	72.86	73.80	74.60	74.60	74.60
/e/	DT	74.40	66.20	66.55	64.86	66.55	75.54	66.54	67.03	65.44	67.03
	RF	70.91	70.40	73.36	74.16	71.08	70.44	75.13	76.05	75.48	75.29
	SVM	75.70	67.31	68.17	64.69	67.67	75.03	48.25	46.24	58.67	46.50
	ANN	72.05	69.59	70.13	68.62	68.49	72.22	73.50	73.95	71.83	73.88
/o/	DT	70.91	67.42	67.81	66.55	67.19	75.54	68.26	68.64	67.42	68.11
	RF	74.40	72.16	72.77	73.06	73.58	70.44	75.79	76.02	75.68	76.37
	SVM	75.70	68.87	67.87	67.41	68.58	75.03	48.37	45.81	61.34	46.96
	ANN	72.05	69.48	70.12	70.35	71.35	72.22	73.08	74.57	72.93	74.95

For /a/ with ANOVA-selected features, RF achieves the highest recall, 76.47%, and SVM achieves the highest precision, 77.92%. For vowel /e/, RF shows improved recall when using IG 76.05% and Chi-square 75.13%, respectively. SVM shows high precision, 75.70% with ANOVA. For /o/, RF has the highest recall values, 76.37% with Gini.

The RF classifier achieves the best accuracy for all vowel signals, while the SVM classifier delivers the best F1 score for these vowels, using the selected optimal features for each vowel. ANOVA consistently leads to high precision and recall for SVM across all vowel classes, indicating it selects features that align well with SVM’s margin-based classification. Gini and ReliefF are more effective with ANN and DT.

**Fig 7:**
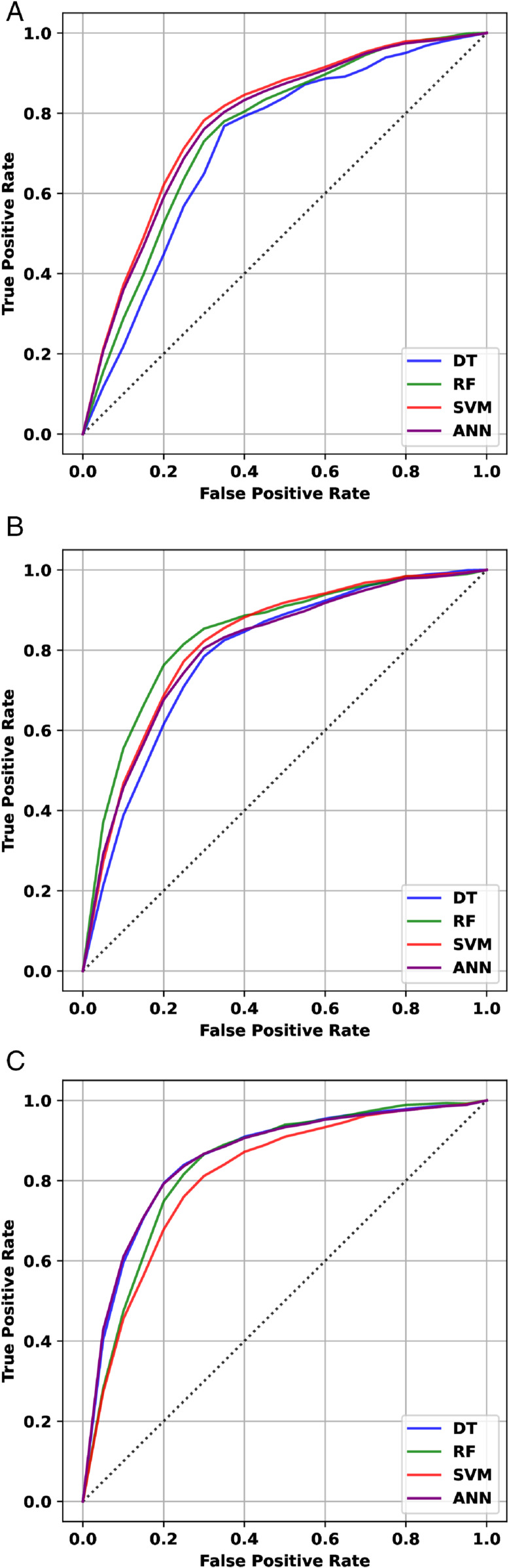
ROC curves for vowels(a) /a/, (b) /e/, and (c) /o/ using RF classifier with ANOVA-based feature selection.

In Fig 7, the Receiver Operating Characteristic (ROC) curve for vowels using an RF classifier with ANOVA feature selection. The ROC curve shows the trade-off between true positive rate (sensitivity) and false positive rate, and demonstrates the model’s ability to discriminate between classes. AUC value indicates the overall performance of the classifier for this vowel.

**Fig 8:**
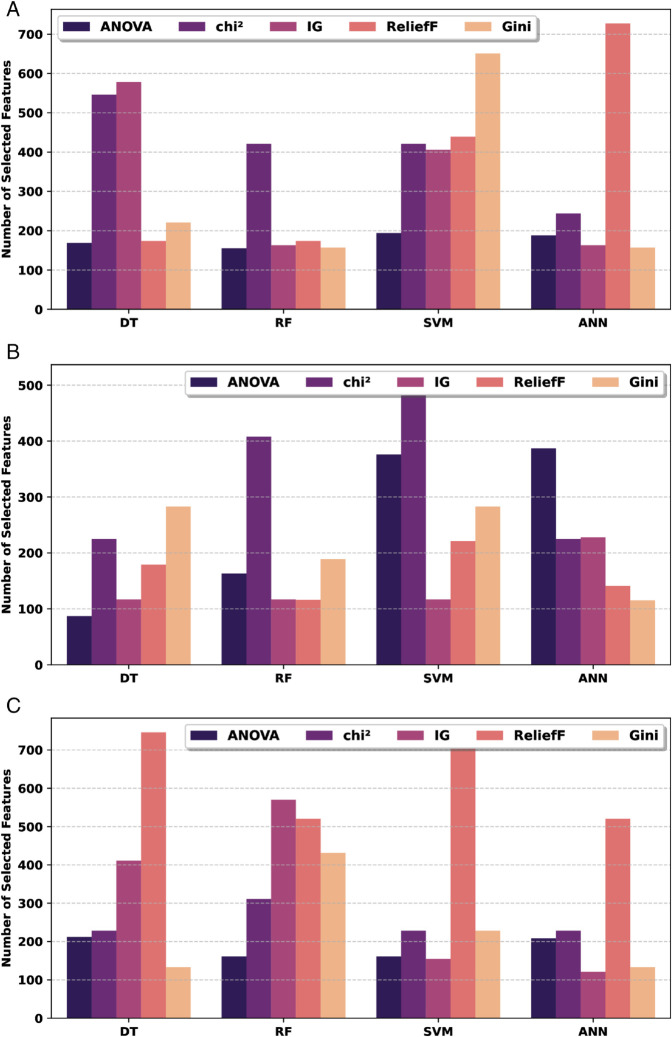
Number of best features selected per model for vowel (a) /a/, (b) /e/, and (c) /o/ signal.

The number of features selected varied significantly across different feature selection techniques and machine learning models, as summarized in Fig 8. For vowel /a/, the highest number of features was 727 selected using the ReliefF with the ANN model, while the fewest were selected, 155, by the ANOVA method with the RF model. For vowel /e/, the chi-square-SVM combination used the highest feature count, whereas the ANOVA-RF selected relatively fewer features, 87. In the case of vowel /o/, the ReliefF-DT combinations selected the largest number of features, 746. ReliefF and Gini methods frequently selected higher numbers of features across vowels and models. ANOVA methods often resulted in more compact feature subsets, particularly with RF models.

**Fig 9:**
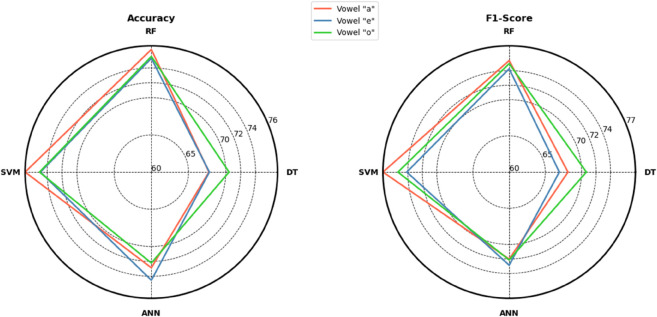
Accuracy and F1-Score of different Models for Vowels /a/, /e/ and /o/.

Accuracy and F1-score comparison of different models is shown in Fig 9. It shows RF and SVM work well for the classification of COVID-19 vowel sound features.

The Friedman test was conducted to statistically compare the performance rankings of the models across the different feature selection methods. Table 5 presents the Friedman ranks of four ML models as evaluated across five feature selection techniques for the vowels. The RF consistently achieved the best performance, with the lowest average rank of 1.2, for vowel /a/ and /o/, and 1.0 for vowel /e/ ranking indicates that the RF model consistently performed better across most feature selection methods.

**Table 5:** Friedman rankings of ML models across feature selection methods for Vowels /a/, /e/, and /o/.

Vowel	Feature Selection	DT	RF	SVM	ANN
/a/	ANOVA	4.0	2.0	1.0	3.0
	chi^2^	3.0	1.0	4.0	2.0
	IG	3.0	1.0	4.0	2.0
	ReliefF	3.0	1.0	4.0	2.0
	Gini	3.0	1.0	4.0	2.0
	**Avg. Rank**	**3.2**	**1.2**	**3.4**	**2.2**
/e/	ANOVA	4.0	1.0	2.0	3.0
	chi^2^	3.0	1.0	4.0	2.0
	IG	3.0	1.0	4.0	2.0
	ReliefF	3.0	1.0	4.0	2.0
	Gini	3.2	1.0	4.0	2.0
	**Avg. Rank**	**3.2**	**1.0**	**3.6**	**2.2**
/o/	ANOVA	4.0	1.0	2.0	3.0
	chi^2^	3.0	2.0	4.0	1.0
	IG	3.0	1.0	4.0	2.0
	ReliefF	3.0	1.0	4.0	2.0
	Gini	3.0	1.0	4.0	2.0
	**Avg. Rank**	**3.2**	**1.2**	**3.6**	**2.0**

**Fig 10:**
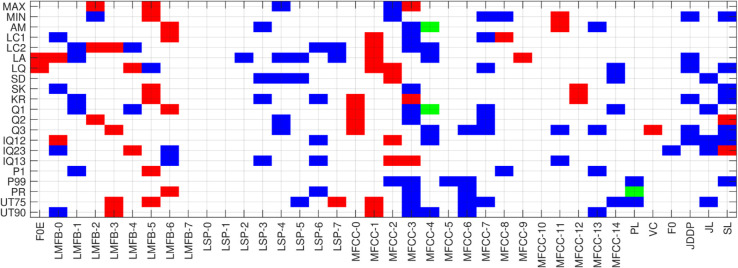
Feature mapping for vowel-a.

**Fig 11:**
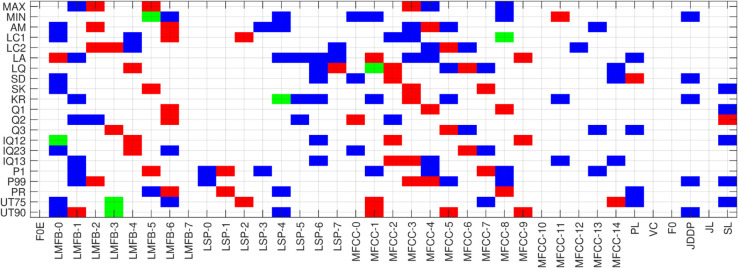
Feature mapping for vowel-e.

**Fig 12:**
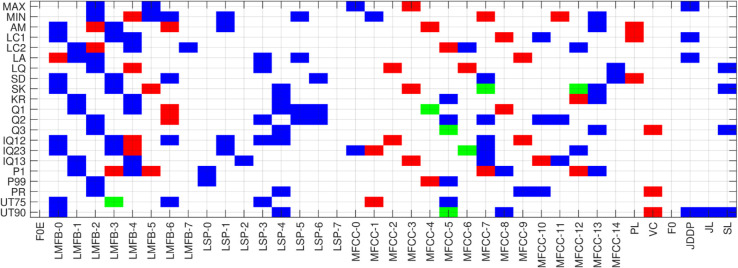
Feature mapping for vowel-o.

The optimal k-features for each vowel across different classification models are identified. Selected features and their mapping functions, categorized by vowel sounds /a/, /e/, and /o/, are presented in Figs 10, 11,12. A map of selected features corresponding and their derivatives (up to a certain degree) is generated using vowel sounds. The horizontal and vertical axes describe 35 features and 21 functions. Color coding is used to indicate the selected features:

- Green color indicates the selection of a functional applied to both features and their derivatives.
- Red color marks selection of a functional applied to only the features.
- Color represents the selection of a functional applied to only the derivative of features.

In the feature selection map in Fig 10 of vowel /a/, 111 features were selected, with 49 being functional features and the remaining 57 functional feature derivatives. This feature map highlights the highest importance assigned to the MFCC (1-4), JDDP, JL, and SL, while FOE, LMFB-7, LSP (0-2), MFCC (9-12), VC, and F0 are of no importance. The remaining features show moderate importance for the classification of COVID-19 using the vowel /a/ signal. Feature selection map for vowel /e/, as shown in Fig 11, selects 116 features, of which 56 are functional of features and the remaining 53 are functional of feature derivatives. This feature map highlights the highest importance assigned to the LMFB-(0-1 and 6), MFCC-(1-8), while FOE, LMFB-7, LSP-(0-2), MFCC-(9-12), VC, and F0 are of the lowest importance. The remaining features show moderate importance for the classification of COVID-19 using the vowel /e/ signal. Feature selection map for vowel /o/, as shown in Fig 12, selects 150 features, of which 37 are functional features and the remaining 106 are functional of feature derivatives. This feature map highlights the highest importance assigned to the LMFB-(0-4 and 6), LSP-(3 and 4), and MFCC-(5 and 7) while FOE, LMFB-7, LSP-(0-3) and LSP-(5-7), MFCC-(5, 7 and 14), PL, VC, FO, JDDP, JL, and SL are of the no importance. The remaining features show moderate importance for the classification of COVID-19 using the vowel /o/ signal.

The comparative analysis of feature selection maps for vowels /a/, /e/, and /o/ in COVID-19 classification reveals both shared and unique feature importance across the vowels. Each vowel had a different total of selected features, with /o/ having the most (150), indicating a greater complexity in relevant patterns, particularly in feature derivatives. Furthermore, the consistently low importance of features such as FOE, LMFB-7, LSP-(0-2), MFCC-(9-12), VC, and F0 suggests that certain higher-order spectral details or fundamental frequency measurements might be less relevant for capturing COVID-19-related changes in these vowels. However, the variations in unimportant features in /o/ suggest that certain MFCCs, LSPs, and additional features like JDDP, JL, and SL have context-specific relevance, which could be more variable in the vowel /o/ compared to /a/ and /e/.

In [9], a smaller subset of samples from the Coswara database was used without detailed filtering or segmentation, which may have included samples with varying recording conditions and quality. Verde *et al*. do not report using a dedicated validation set, which increases the risk of overfitting and may inflate the reported accuracy. This study focuses on a larger subset comprising sustained vowel sounds (/a/, /o/, and /e/). Third, feature selection in our study is deliberately focused and explainable. The feature selection uses all acoustic features using OpenSMILE, avoiding feature overloading or black-box feature learning.

In the study, a few limitations need to be acknowledged. The feature extraction process relied entirely on hand-crafted features using the OpenSMILE toolkit. Second, the performance of the classifiers showed sensitivity to the chosen feature selection methods. Although statistical validation using Friedman and post hoc tests was performed, model behavior may vary with different feature engineering pipelines or when applied to unseen datasets. The dataset used in this study, while informative, may have limitations in terms of speaker diversity, recording conditions, or representation across demographics, which could affect generalizability.

For future work, we plan to explore deep end-to-end learning models that can learn feature representations directly from raw audio. Expanding the dataset with more diverse speech samples and implementing advanced optimization strategies can further enhance model robustness. Additionally, incorporating explainable AI (XAI) techniques could offer better transparency into model decisions, which is critical for clinical and diagnostic applications.

## 4 Conclusion and future works

This study demonstrated the effectiveness of respiratory sound analysis using OpenSMILE-derived features combined with machine learning classifiers for the detection of COVID-19. Among the evaluated models, the Random Forest (RF) classifier consistently performed well for all vowels, achieving accuracies of 76.47%, 75.54%, and 75.54% for vowel /a/, /e/, and /o/, respectively. The ANOVA-based feature selection method effectively reduced dimensionality by selecting smaller subsets of salient features—specifically 155, 163, and 161 features for vowels /a/, /e/, and /o/, respectively—while maintaining competitive classification performance. To assess the statistical significance of classifier performance across vowels, the Friedman test was conducted, revealing significant differences (*p*<0.05), thereby validating the robustness of the RF classifier. These findings underscore the importance of both targeted feature selection and classifier choice in respiratory sound analysis.

## References

[1] Al-Khassaweneh M, Bani Abdelrahman R. A signal processing approach for the diagnosis of asthma from cough sounds. J Med Eng Technol. 2013;37(3):165–71. doi: 10.3109/03091902.2012.758322 23631519

[2] Al-Shourbaji I, Kachare PH, Abualigah L, Abdelhag ME, Elnaim B, Anter AM, et al. A deep batch normalized convolution approach for improving COVID-19 detection from chest X-ray images. Pathogens. 2022;12(1):17. doi: 10.3390/pathogens12010017 36678365 PMC9860560

[3] Deshpande G, Schuller BW. COVID-19 biomarkers in speech: on source and filter components. Annu Int Conf IEEE Eng Med Biol Soc. 2021;2021:800–3. doi: 10.1109/EMBC46164.2021.9629831 34891411

[4] Rao S, Narayanaswamy V, Esposito M, Thiagarajan JJ, Spanias A. COVID-19 detection using cough sound analysis and deep learning algorithms. IDT. 2022;15(4):655–65. doi: 10.3233/idt-210206

[5] Khan TA, Vijayakumar P. Separating heart sound from lung sound UsingLabVIEW. International Journal of Computer and Electrical Engineering. 2010;2(3):524.

[6] Pahar M, Klopper M, Warren R, Niesler T. COVID-19 cough classification using machine learning and global smartphone recordings. Comput Biol Med. 2021;135:104572. doi: 10.1016/j.compbiomed.2021.104572 34182331 PMC8213969

[7] Pahar M, Klopper M, Warren R, Niesler T. COVID-19 detection in cough, breath and speech using deep transfer learning and bottleneck features. Comput Biol Med. 2022;141:105153. doi: 10.1016/j.compbiomed.2021.105153 34954610 PMC8679499

[8] Wall C, Zhang L, Yu Y, Kumar A, Gao R. A deep ensemble neural network with attention mechanisms for lung abnormality classification using audio inputs. Sensors (Basel). 2022;22(15):5566. doi: 10.3390/s22155566 35898070 PMC9332569

[9] Verde L, De Pietro G, Ghoneim A, Alrashoud M, Al-Mutib KN, Sannino G. Exploring the use of artificial intelligence techniques to detect the presence of coronavirus Covid-19 through speech and voice analysis. IEEE Access. 2021;9:65750–7. doi: 10.1109/ACCESS.2021.3075571 35256922 PMC8864957

[10] Abayomi-Alli OO, Damaševičius R, Abbasi AA, Maskeliūnas R. Detection of COVID-19 from deep breathing sounds using sound spectrum with image augmentation and deep learning techniques. Electronics. 2022;11(16):2520. doi: 10.3390/electronics11162520

[11] Liu S, Mallol-Ragolta A, Schuller BW. COVID-19 detection from speech in noisy conditions. In: ICASSP 2023 - 2023 IEEE International Conference on Acoustics, Speech and Signal Processing (ICASSP). 2023. p. 1–5. 10.1109/icassp49357.2023.10094304

[12] Imran A, Posokhova I, Qureshi HN, Masood U, Riaz MS, Ali K, et al. AI4COVID-19: AI enabled preliminary diagnosis for COVID-19 from cough samples via an app. Inform Med Unlocked. 2020;20:100378. doi: 10.1016/j.imu.2020.100378 32839734 PMC7318970

[13] Bhattacharya D, Sharma NK, Dutta D, Chetupalli SR, Mote P, Ganapathy S, et al. Coswara: a respiratory sounds and symptoms dataset for remote screening of SARS-CoV-2 infection. Sci Data. 2023;10(1):397. doi: 10.1038/s41597-023-02266-0 37349364 PMC10287715

[14] Xu Z, Strake M, Fingscheidt T. Deep noise suppression maximizing non-differentiable PESQ mediated by a non-intrusive PESQNet. IEEE/ACM Trans Audio Speech Lang Process. 2022;30:1572–85. doi: 10.1109/taslp.2022.3165442

[15] Kumar Shukla N, Shajin FH, Rajendran R. Speech enhancement system using deep neural network optimized with Battle Royale Optimization. Biomedical Signal Processing and Control. 2024;92:105991. doi: 10.1016/j.bspc.2024.105991

